# The practical roadmap for peri-cystectomy approaches in muscle-invasive bladder cancer

**DOI:** 10.3389/fonc.2025.1543837

**Published:** 2025-04-10

**Authors:** Joseph Kattan, Clarisse Kattan, Fouad Aoun, Elie Nemr

**Affiliations:** ^1^ Department of Hematology-Oncology, Hotel-Dieu de France University Hospital, Faculty of Medicine, Saint Joseph University of Beirut, Beirut, Lebanon; ^2^ Department of Urology, Hotel-Dieu de France University Hospital, Faculty of Medicine, Saint Joseph University of Beirut, Beirut, Lebanon

**Keywords:** muscle invasive bladder cancer, neo-adjuvant chemotherapy, neo-adjuvant chemoimmunotherapy, adjuvant chemotherapy, adjuvant immunotherapy, peri-cystectomy therapy

## Abstract

The management of muscle-invasive bladder cancer (MIBC) remains a challenging topic since it is witnessing rapidly evolving changes and new drug approvals. In fact, more effective approaches are now available to improve the outcomes of patients with MIBC who are candidates for cystectomy. Neo-adjuvant cisplatin-based chemotherapy was the standard approach for patients who were deemed cisplatin-eligible. Also, adjuvant cisplatin-based chemotherapy was considered for high-risk operated patients who did not receive the standard neo-adjuvant chemotherapy. It was only recently that adjuvant immune checkpoint inhibitors were proved effective in adjuvant settings and were approved for high-risk MIBC patients after neo-adjuvant chemotherapy followed by cystectomy or for those who did not receive neo-adjuvant chemotherapy and were not eligible for adjuvant cisplatin-based chemotherapy. More recently, adding immune checkpoint inhibitors to neo-adjuvant chemotherapy and to post-cystectomy adjuvant therapy seemed to be very promising. In this review article, all current peri-cystectomy options are briefly described with an attempt to guide and simplify choices by drawing a roadmap covering all the practical scenarios.

## Introduction

Almost 75% of patients with bladder cancer have a non-muscle-invasive tumor associated with a good prognosis. However, half of these patients will experience recurrence.

The remaining 25% of patients have muscle-invasive or metastatic bladder cancer at presentation, associated with a poor prognosis with a 15% overall survival rate at 5 years (1).

Muscle-invasive bladder cancer (MIBC) has been recently witnessing practical evolution, making the management of these patients challenging for oncologists. As a matter of fact, in early-stage MIBC, almost half of the patients treated with curative radical cystectomy relapsed with 50% to 60% overall survival at 5 years (2, 3).

To enhance patients’ outcomes, cystectomy was no longer considered alone as the standard of care and became associated with either neo-adjuvant or adjuvant chemotherapy. More recently, immune checkpoint inhibitors (ICIs) were added to the therapeutic arsenal and were introduced in either the pre- or post-operative setting or in both.

In this brief review, a simplified roadmap will be drawn to help define the adequate associated therapy, either before or after surgery, according to the convincing data available in the literature today.

## Neo-adjuvant chemotherapy

Until recently, the standard approach for patients with MIBC was neo-adjuvant chemotherapy (NACT) followed by radical cystectomy. This long-lasting practice was based on the results of a meta-analysis of randomized studies from the literature (3, 4) where NACT reduced death by 20% with a 5% absolute life gain during a 10-year period.

These studies focused on cisplatin-based NACT with either gemcitabine (GC) or dose-dense methotrexate, vinblastine, adriamycin, and cisplatin (MVAC). Recently, the French VESPER randomized trial showed a better outcome when dose-dense MVAC (dd-MVAC) was used compared to GC (5, 6), with a higher local control rate (complete pathological response or tumor downstaging) in the dd-MVAC arm [pathological complete response (pCR) of 42% versus 36% with *p* = 0.021]. Additionally, overall survival at 5 years was improved in the dd-MVAC group versus the GC group [66% *vs*. 57%, hazard ratio (HR) = 0.71], as well as the time to death due to bladder cancer (5-year cumulative incidence, 24% *vs*. 38%, HR = 0.55) (7).

Therefore, patients with MIBC who are candidates for radical cystectomy must be fit for cisplatin-based NACT (either with GC or with dd-MVAC) as an imperative condition to undergo NACT as the most effective and recommended approach. These patients have to fulfill all these criteria to be deemed fit for NACT: performance status <2, creatinine clearance >60 mL/min, good cardiac function with left ventricular ejection fraction (LVEF) > 50%, and absence of severe peripheral neuropathy or hearing loss (8).

Patients who do not meet these criteria will not be eligible for NACT and will undergo an upfront cystectomy. Interestingly, omitting cisplatin or replacing it with carboplatin in the NACT is not an accepted alternative.

Pathological complete response assessed on the cystectomy material after cisplatin-based NACT is observed in approximately 40% of the treated patients (6, 9). It is almost confirmed that pathological response predicts overall survival in these patients since a retrospective study of 2,010 patients from the National Cancer Database showed that the 5-year overall survival rate for patients who achieved pathological downstaging and pathological complete response were 70% and 84%, respectively (10).

## Adjuvant chemotherapy

Data from the old literature were always considered not strong enough to recommend adjuvant chemotherapy for those who did not receive NACT. The results of these phase II studies were too variable to provide strong recommendations on its use, even if there was a favorable trend toward using adjuvant chemotherapy (11). However, a more updated meta-analysis including 10 randomized controlled trials demonstrated the benefit of adjuvant cisplatin-based chemotherapy on overall survival (HR = 0.82, 95% CI = 0.70–0.96, *p* = 0.02) with an absolute improvement in survival of 6% at 5 years and a 9% absolute benefit when adjusted for age, sex, pT stage, and pN category (HR = 0.77, 95% CI = 0.65–0.92, *p* = 0.004). Adjuvant chemotherapy also demonstrated improvement in recurrence-free survival (HR = 0.71, 95% CI = 0.60–0.83, *p* < 0.001), locoregional recurrence-free survival (HR = 0.68, 95% CI = 0.55–0.85, *p* < 0.001), and metastasis-free survival (HR = 0.79, 95% CI = 0.65–0.95, *p* = 0.01), with absolute benefits of 11%, 11%, and 8%, respectively (12).

Nowadays, the National Comprehensive Cancer Network (NCCN) guidelines recommend adjuvant chemotherapy in high-risk MIBC post-cystectomy patients (pT3, pT4a, or pN+) who did not receive NACT. In this setting, adjuvant cisplatin-based chemotherapy is the preferred therapeutic option (13).

## Neo-adjuvant chemo-immunotherapy

Recently, the immunochemotherapy neo-adjuvant approach by adding ICIs to NACT was investigated and seemed promising. Eighty-one patients received five cycles of pembrolizumab added to either GC for cisplatin-eligible patients or to gemcitabine alone for ineligible patients in a phase Ib/II, open-label, single-arm study. The pathological muscle-invasive response rate was 54% in cisplatin-eligible patients with 41% of patients downstaged to a pathological complete response compared to 53% pathological muscle-invasive response rate in the cisplatin-ineligible patients of whom 41% were downstaged to pathological complete response rate. Of all patients, the 18-month relapse-free survival was 65.1%, and the 3-year overall survival (OS) was 65.7%. Neo-adjuvant chemo-immunotherapy with pembrolizumab showed significant pathological downstaging in patients who are cisplatin-eligible and cisplatin-ineligible (14).

Also, nivolumab every 2 weeks was added to four cycles of cisplatin plus gemcitabine NACT in 49 patients. The clinical complete response was 59% after completion of neo-adjuvant therapy, and the pCR was 35% among the 34 cystectomized patients. The median disease-free survival (DFS) was not reached (15).

Likewise, avelumab was assessed with NACT in a randomized phase II trial where patients were separated into two groups and randomized to either GC or dd-MVAC for the cisplatin-eligible cohort and to paclitaxel-gemcitabine or avelumab monotherapy for the cisplatin-ineligible cohort. In the cisplatin-eligible cohort, pCR was observed in 22/38 (58%) patients in the dd-MVAC + avelumab arm and 19/35 (54%) patients in the GC + avelumab arm. The 12-month event free survival (EFS) rates were 92% in the dd-MVAC + avelumab arm, compared to 84% for GC + avelumab. For overall survival, the 12-month rates were slightly higher in favor of dd-MVAC + avelumab (95% versus 92%) and considerably higher at 36 months in favor of dd-MVAC + avelumab (85% versus 64%). In the cisplatin-ineligible cohort, pCR was observed in only 4/28 (14%) and 9/27 (33%) patients in the paclitaxel + gemcitabine + avelumab and avelumab monotherapy arms, respectively. The event-free and overall survival rates were similar between the two arms (16).

Moreover, the combination of four cycles of dd-MVAC plus four doses of durvalumab showed a 71% pathological downstaging with 49% pCR among 55 patients who underwent cystectomy. However, adding tremelimumab to durvalumab did not show any added benefit (17, 18).

Another single-arm phase II study associated durvalumab with GC in a peri-cystectomy setting in 61 patients. After the four cycles of NACT plus durvalumab, complete pathological response in resected patients was achieved in 17 patients (33%), and 31 (60%) had pathological response <ypT2 ypN0 (18).

## Adjuvant immunotherapy

Since 2021, the results of three randomized phase III trials comparing three different ICIs in an adjuvant setting versus observation or placebo have been reported consecutively (**Table 1**). These studies evaluated 1 year of either atezolizumab (IMvigor010), nivolumab (CheckMate 274), or pembrolizumab (Ambassador). MIBC patients with a high risk of recurrence after radical surgery were selected according to almost the same criteria in all these three studies and included either ypT2-4a or ypN-positive patients who had prior neo-adjuvant cisplatin-based NACT, or pT3-4a or pN-positive patients without prior neo-adjuvant cisplatin-based NACT and not eligible for or who refused adjuvant cisplatin-based NACT.

**Table 1:** Results of the three phase III trials of adjuvant immunotherapy in MIBC.

Phase III trial (ref)	Adjuvant ICI	Number of patients	DFS, HR (95% CI)	OS, HR (95% CI)
IMvigor010 (19)	Atezolizumab	809	0.89 (0.74–1.08)	0.91 (0.73–1.13)
CheckMate 274 (20)	Nivolumab	709	0.71 (0.58–0.86)	0.76 (0.61–0.96)
Ambassador (21)	Pembrolizumab	702	0.69 (0.54–0.87)	Not yet mature

ICI, immune checkpoint inhibitor; DFS, disease-free survival; HR, hazard ratio; CI, confidence interval; OS, overall survival; MIBC, muscle-invasive bladder cancer.

The first reported study was IMvigor010, which compared 1-year adjuvant atezolizumab (an anti-PDL1) to observation. The trial did not meet its primary endpoint of improved disease-free survival in the atezolizumab group and high frequencies of adverse events leading to the discontinuation of atezolizumab were reported. Therefore, the data did not support the use of this ICI in the adjuvant setting (19).

The second reported trial was CheckMate 274, where patients with MIBC who underwent radical cystectomy were randomized to receive 1 year of either nivolumab, an anti-PD1, or placebo. DFS results were positive with a doubled median DFS compared to placebo (20.8 months with nivolumab versus 10.8 months with placebo). Disease-free survival was more pronounced in patients with tumor PDL1 expression ≥1%, with an HR of 0.55 in this subgroup *vs*. 0.70 in the intention-to-treat population. Also, interim OS data favored nivolumab versus placebo in the intent-to-treat population [69.5 *vs*. 50.1 months with HR = 0.76 (0.61–0.96)]. Treatment-related adverse events occurred in 17.9% of the nivolumab group and 7.2% of the placebo group. Consequently, adjuvant nivolumab in MIBC earned the Food and Drug Administration (FDA) approval on August 19, 2021, regardless of the PDL1 status (20).

The last reported randomized study was Ambassador, which compared 1 year of adjuvant pembrolizumab, anti-PD1, to observation. DFS was significantly improved with a median of 29.6 months versus 14.2 with HR = 0.73 (0.59–0.90). Interim OS analysis showed a median OS of 50.9 months with pembrolizumab *vs*. 55.8 months with observation (HR = 0.98, 95% CI = 0.76–1.26, *p* = 0.88). Final results were not reported, and the FDA approval was not guaranteed yet (21).

## Peri-cystectomy chemo-immunotherapy

Perioperative durvalumab was shown to be safe and efficacious in a phase II study, where durvalumab was added to four cycles of GC in a neo-adjuvant setting in 61 patients with MIBC, followed by 10 cycles of durvalumab in the adjuvant setting. In addition to pCR in 33% and pathological response (<ypT2 ypN0) in 60%, OS reached 85% at 2 years and 81% at 3 years (18).

Recently, based on these encouraging results of phase II peri-operative durvalumab, Powles T. et al. reported their positive phase III study where 1,063 patients with MIBC were randomized to receive either NACT with durvalumab added to four cycles of GC followed by 8 cycles of adjuvant durvalumab alone, or the comparator arm, which included the four cycles of neo-adjuvant GC alone. This study, called NIAGARA, demonstrated a statistically significant and clinically meaningful improvement in event-free survival (HR = 0.68, 95% CI = 0.56–0.82, *p* < 0.0001) and OS (HR = 0.75, 95% CI = 0.59–0.93, *p* = 0.0106), with a similar percentage of grade 3 or 4 treatment-related adverse events in both groups. The NIAGARA supported perioperative durvalumab with NACT as a potential new standard treatment for patients with cisplatin-eligible MIBC (22).

## Practical guidance and roadmap

The aim of our review was to draw a simplified roadmap based on the new up-to-date evidence to help physicians in their decision-making when treating patients diagnosed with MIBC. Before drawing the roadmap according to real-world patient scenarios, some rules must be respected when applying peri-cystectomy approaches in MIBC patients:

- Carboplatin should not be used in neo- or adjuvant chemotherapy since neo-adjuvant or adjuvant chemotherapy recommendations are based only on cisplatin.
- Adjuvant chemotherapy should not be used (post-cystectomy) if neo-adjuvant cisplatin-based chemotherapy was given.
- Indications for adjuvant ICIs are limited to those of high risk who did not receive cisplatin chemotherapy either as neo-adjuvant or adjuvant, and for those after neo-adjuvant cisplatin-based chemotherapy when histology shows persistent high-risk features (ypT2-T4a or ypN+).
- Finally, there is no place for adjuvant ICIs after adjuvant cisplatin-based chemotherapy.

**Figure 1:**
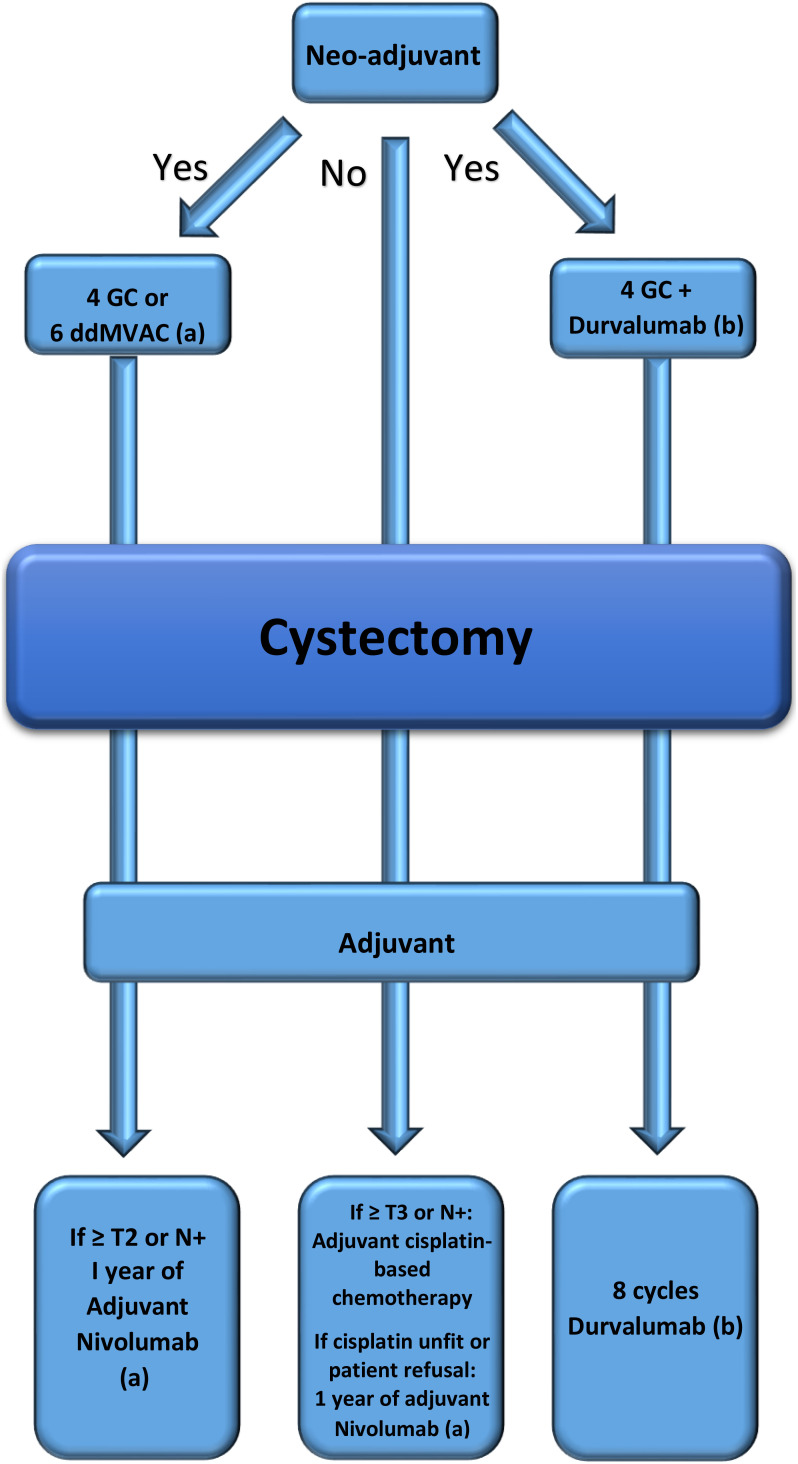
Roadmap drawn according to real-world patient scenarios. **(a)** Pembrolizumab could be an alternative if approved. **(b)** The third scenario is not approved yet.

**Figure 1** summarizes the three different scenarios of patients undergoing cystectomy for MIBC where adjuvant or neo-adjuvant therapies are adequately attributed.
